# Preoperative prediction of microvascular invasion in pancreatic neuroendocrine tumors through analysis of portal venous phase CT images

**DOI:** 10.1186/s13244-025-02091-6

**Published:** 2025-09-25

**Authors:** Hai-Yan Chen, Yao Pan, Yu-Wei Li, Li-Ting Shi, Jie-Yu Chen, Yun-Ying Liu, Ri-Sheng Yu, Lei Shi

**Affiliations:** 1 Department of Radiology, Zhejiang Cancer Hospital, Hangzhou Institute of Medicine (HIM), Chinese Academy of Sciences, Hangzhou, China; 2 Department of Radiology, Second Affiliated Hospital, Zhejiang University School of Medicine, Hangzhou, China; 3 Department of Pathology, Zhejiang Cancer Hospital, Hangzhou Institute of Medicine (HIM), Chinese Academy of Sciences, Hangzhou, China

**Keywords:** Pancreas, Neuroendocrine tumors, Microvascular invasion, Survival analysis, Shiny

## Abstract

**Objectives:**

To evaluate clinical and CT imaging features on portal venous-phase for predicting microvascular invasion (MVI) in patients with pancreatic neuroendocrine tumors (PNETs) and compare survival outcomes.

**Materials and methods:**

In this retrospective study, 160 patients (training group) and 28 (validation group) who underwent surgical resection for PNETs were included. Demographic data and CT features were collected. The independent predictive factors for predicting MVI were confirmed through univariate and multivariate logistic regression analyses. The predictive performance was assessed by employing the receiver operating characteristic curve for predicting MVI. An R/shiny app based on logistic regression was developed. A Kaplan-Meier survival analysis with a log-rank test was conducted.

**Results:**

In the training group, invasion of surrounding tissues (odds ratio [OR]: 4.12), absolute enhancement (OR: 0.84), and relative enhancement ratio (OR: 16.1) were identified as independent predictors for predicting MVI in PNET patients, with an area under the curve of 0.819 and 0.891 in the training and validation groups, respectively. We have successfully developed a user-friendly web-based R/shiny app for real-time prediction of MVI in patients with PNETs. The median overall survival for patients with MVI was 12 months, compared to 37.5 months for those without MVI (log-rank *p* = 0.034).

**Conclusions:**

Imaging features from portal venous-phase CT images can be used to accurately predict the presence of MVI in patients with PNETs. Patients with MVI are associated with worse survival compared to those without MVI. The web-based R/shiny app for predicting MVI provides real-time data-driven estimates of predictive value to facilitate informed decision-making.

**Critical relevance statement:**

Imaging features can accurately predict MVI in patients with PNETs, and the web-based R/shiny app provides real-time, data-driven estimates to enhance decision-making, thereby streamlining clinical practice.

**Key Points:**

- The presence of microvascular invasion (MVI) in patients was associated with worse survival.
- Surrounding tissue invasion and absolute/relative enhancement ratio were identified as independent predictors for MVI.
- This web-based app predicts MVI and provides real-time data-driven estimates of predictive value.

**Graphical Abstract:**

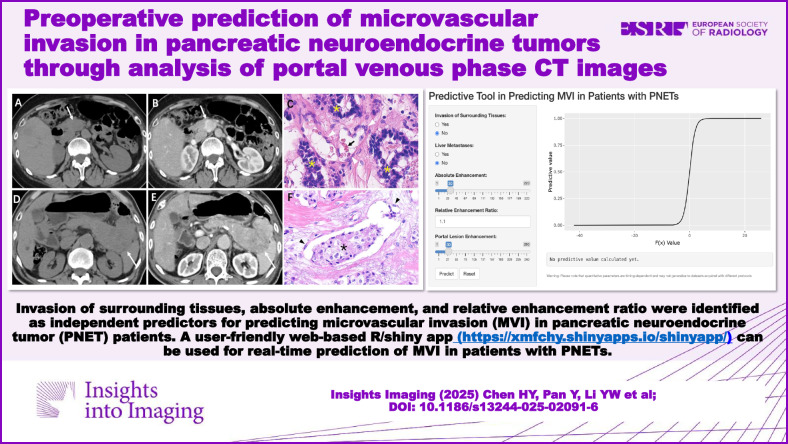

## Introduction

The incidence of pancreatic neuroendocrine tumors (PNETs), a highly heterogeneous group of tumors, has increased due to widespread CT use and better early detection [1]. The radical operation is generally considered the definitive treatment for localized PNETs [2, 3]. Histopathologic evaluation in PNETs is crucial for prognostication and therapy personalization, linked to specific morphological characteristics [4, 5]. The PNETs are divided into grade 1 (G1), grade 2 (G2), grade 3 (G3) and neuroendocrine carcinomas (NECs) based on Ki-67 index and mitotic count [6]. Furthermore, the biological heterogeneity of PNETs varies greatly from well-differentiated tumors to poorly differentiated tumors, even within small entities [7].

The presence of microvascular invasion (MVI), an important histological parameter, is observed in surgical specimens of PNETs at a prevalence ranging from 14% to 37.6%, which has been identified as a strong independent risk factor for both overall survival and recurrence-free survival, even among grade 1 patients and those who undergo limited operations [8–11]. However, the nature of MVI remains poorly understood, with limited available knowledge. Predictive factors of MVI before operation remain a persistent challenge yet hold significant clinical implications.

Contrast-enhanced CT imaging features have been extensively studied for grading, differentiation, aggressiveness, and survival outcomes of PNETs, but their use in predicting MVI is largely unexplored [12–16]. MVI, along with tumor diameter > 20 mm, perineural invasion, and distant metastases, were identified as independent risk factors correlated with lymph node metastases [17]. Meanwhile, MVI was also associated with the Ki-67 index, providing insights into tumor heterogeneity to a certain extent [18]. CT imaging features have not been used to predict MVI, warranting further investigation for better clinical decisions. Deploying models in practice is challenging. The shiny app, developed in R, provides an interactive interface for real-time data visualization, disease prediction, and decision-making, and has been used in small-scale studies [19, 20], with no existing reports specifically addressing PNETs.

The identification of qualitative features from portal venous phase images has proven to be a valuable parameter in the analysis of prognosis and differential diagnosis [16, 21, 22]. Therefore, the objective of this current multicenter population-based study was to utilize clinical and imaging features from portal venous phase CT images to accurately predict the presence of MVI before surgery. Additionally, we have developed a web-based R/shiny app for predicting MVI in PNETs using clinical parameters. Survival analysis among different PNET patient subgroups was also conducted.

## Materials and methods

The retrospective study was approved by the Ethics Committee of the Second Affiliated Hospital of Zhejiang University School of Medicine (Approval number: 2021-0554) and Zhejiang Cancer Hospital (Approval number: IRB-2024-1321), which waived the need for written informed consent.

### Study population

All patients in our study had surgically confirmed PNETs. The training data consisted of consecutive patients with pathologically confirmed PNETs, retrospectively analyzed at institution 1 (Second Affiliated Hospital, Zhejiang University School of Medicine) from January 2010 to December 2022 (*n* = 130), and at institution 2 (Zhejiang Cancer Hospital) from January 2009 to March 2023 (*n* = 30), respectively. Additionally, the validation group was collected from January 2023 to December 2023 at institution 1 (*n* = 21), and from April 2023 to April 2024 at institution 2 (*n* = 7). It is worth mentioning that the data of our validation group was collected after the establishment of the training group model; this approach has a similar effect to prospective validation.

**Fig. 1:**
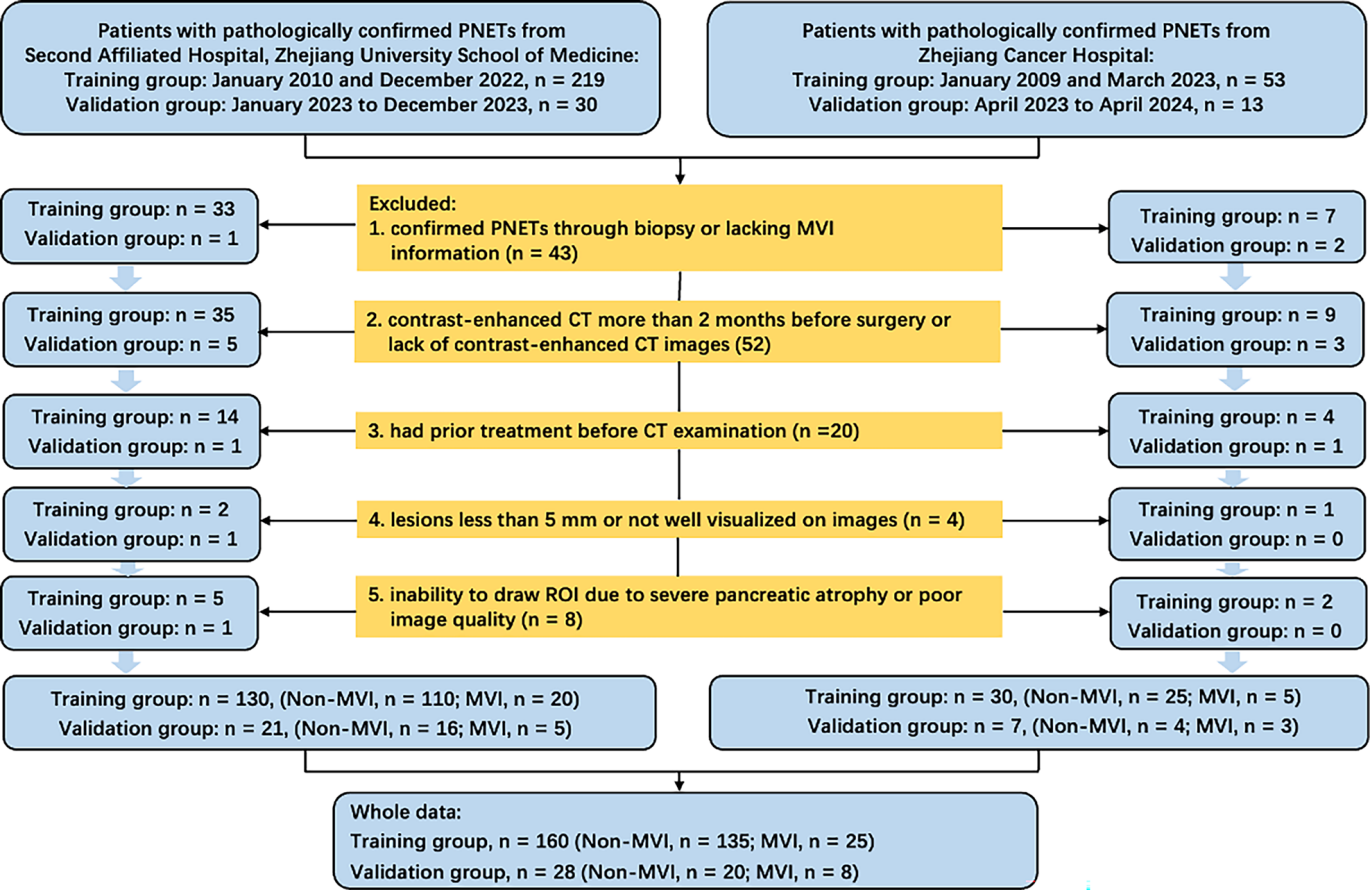
The flowchart illustrating the criteria for patient inclusion and exclusion in the study

The inclusion criteria were as follows: (1) confirmed PNETs via surgery with MVI status in pathology; (2) contrast-enhanced CT within 2 months before surgery; (3) no prior treatment before CT. The exclusion criteria were as follows: (1) PNETs confirmed by biopsy or lacking MVI info; (2) lesions too small or not visible; (3) ROI delineation not possible due to severe atrophy or poor image quality. In patients with multiple lesions, only the largest one was included for further analysis. Finally, a total of 160 patients (Non-MVI, *n* = 135, MVI, *n* = 25) were included in the training group, and 28 patients (Non-MVI, *n* = 20, MVI, *n* = 8) in the validation group were included (Fig. 1). The average duration between CT scans and surgery was 8 days, ranging from 1 to 59 days.

### CT techniques

Due to the prolonged duration of data collection, multiple CT protocols were used (Supplementary Material 1). The CT images were acquired using multidetector row systems with a minimum of 16 detectors. The agent was administered intravenously using an automatic power injector at a rate of 3 mL/s. The portal venous phase images were acquired 45–60 s after the contrast injection, with most scans performed at approximately 60 s. This timing was selected to ensure optimal contrast enhancement of the portal vein and surrounding structures, while also balancing the demands of a heavy clinical workload and maintaining efficiency. The images were obtained using a protocol that utilized 120 kVp and 150–350 mAs, with a slice thickness ranging from 3.0 to 5.0 mm. Additionally, for images obtained after 2020, 1.25 mm thin-slice reconstructions were performed during the portal venous phase.

### Image analysis

The image features were evaluated by two abdominal radiologists (with 6 and 8 years of experience in pancreatic radiology diagnosis) with a consensus who were unaware of the pathology and possessed expertise in this field.

### Qualitative analysis

Demographic information, including age, gender, symptoms, and tumor markers, was collected. Tumor marker elevation was defined as an increase in carbohydrate antigen 19-9 or 25, or carcinoembryonic antigen. Overall survival was the time from surgery to death. Censored data included patients still alive at the last follow-up (April 1, 2024) or lost to follow-up without explanation. Due to the validation group’s short follow-up, only the training group survival data were used. In addition, functional status and tumor grade were described as additional information.

The qualitative factors were listed as follows: tumor location (head/neck vs. body/tail), pancreatic duct dilatation (main duct diameter ≥ 3 mm [14]), calcification, shape (round vs. irregular), tumor texture (solid vs. solid and cystic), invasion of surrounding tissues (involved adjacent tissues/organs/vessels, including venous thrombus and vessel occlusion), lymph nodes metastases (short axis larger than 1 cm, with abnormal round morphology or necrosis within the center [23]), and liver metastases (the presence of multiple peripheral enhanced/hypervascular lesions [24], if available, MRI was used as a reference). A round shape was defined as a tumor with a circular or oval appearance in over 80% of transverse sections, while an irregular shape was a non-circular or angular shape in more than 20% of sections [14]. A solid tumor was defined as having a solid component > 90% of the tumor, while the rest were classified as solid and cystic components. Vascular involvement was assessed by the degree of circumferential contact between the tumor and vessel (> 180°), or irregular vessel contours or caliber changes (including ‘tear drop’ deformity), using pancreatic cancer vascular invasion criteria [23].

Based on the institutional surgical strategy and guideline recommendations [25, 26], for patients with resectable liver metastases, simultaneous surgery for both the primary tumor and liver metastases can be performed. For unresectable cases, primary tumor resection can be considered to reduce tumor burden, taking into account the patient’s preferences.

### Quantitative analysis

All quantitative measures were performed in duplicate, and averages were used for analysis. The largest diameter (mm) was measured on the axial image. Hounsfield Unit (HU) values for the PNET tumor and surrounding normal parenchyma were collected in both the unenhanced and portal venous phases. HU values were obtained by delineating a circular or ovoid ROI at the tumor’s maximum diameter, avoiding calcification, necrosis, cystic, and hemorrhagic areas. A comparable ROI was manually drawn in the nearby normal parenchyma, avoiding the pancreatic duct, vessels, tumor margins and areas affected by pancreatitis or fatty infiltration. The tumor and parenchyma ROIs from the portal venous phase were transferred to the plain phase with placement corrections as needed, ensuring high coincidence between plain and enhanced phase ROIs.

The CT enhanced variables were included as follows: (1) enhancement ratio was calculated as enhanced tumor attenuation/nearby parenchyma attenuation; (2) absolute enhancement was defined as the subtraction of the unenhanced from enhanced tumor HU value; (3) relative enhancement ratio was calculated by dividing the absolute enhancement by the unenhanced tumor HU value (× 100%) [13, 27].

The intraclass correlation coefficient (ICC) was calculated for the validation group (*n* = 28) only selected parameters (portal lesion enhancement, enhancement ratio, absolute enhancement, relative enhancement ratio) to assess interobserver stability and reproducibility. Results were considered stable if ICC > 0.9 [28].

### Statistical analysis

Qualitative data were reported as numbers and percentages, with chi-square or Fisher’s exact test used. Quantitative data were expressed as means ± standard deviations or median (25–75%), with the Student’s *t*-test or Mann–Whitney U test applied. Multivariable logistic regression with backward stepwise selection identified independent predictors for MVI in the training group, including only parameters with *p* < 0.05 from the univariable analysis. Odds ratios (OR) and 95% confidence intervals (CI) were recorded, and a forest plot was generated. Receiver operating characteristic (ROC) analysis assessed the predictive performance of selected factors for MVI, including area under the curve (AUC), sensitivity, and specificity. Sensitivity and specificity thresholds for absolute and relative enhancement ratios were computed. A web-based shiny app (shiny 1.7.5) was developed in R (version 4.1.2) using ‘shiny’, ‘ggplot2’, and ‘plotly’ packages, based on the logistic regression formula. Intraclass correlation coefficient (ICC) was determined with a two-way random model, and 95% CI was calculated. Pearson correlation analysis was performed between the largest diameter and the enhancement parameters. Kaplan–Meier survival analysis with a log-rank test was conducted to compare actual and predicted overall survival. Statistical significance was set at *p* < 0.05. Forest plots were created in R (version 4.1.2), Kaplan–Meier curves and Pearson correlation analysis were presented in Python (version 3.10), and other analyses in SPSS 26.0.

## Results

### Demographic information and radiological characteristics of the training group

First, among the 188 patients, 89 were classified as G1, 82 as G2, 9 as G3, and 8 as NECs. Regarding functional status, 152 patients had non-functional tumors and 36 had functional tumors; however, only one patient in the MVI group had a functional tumor.

**Fig. 2:**
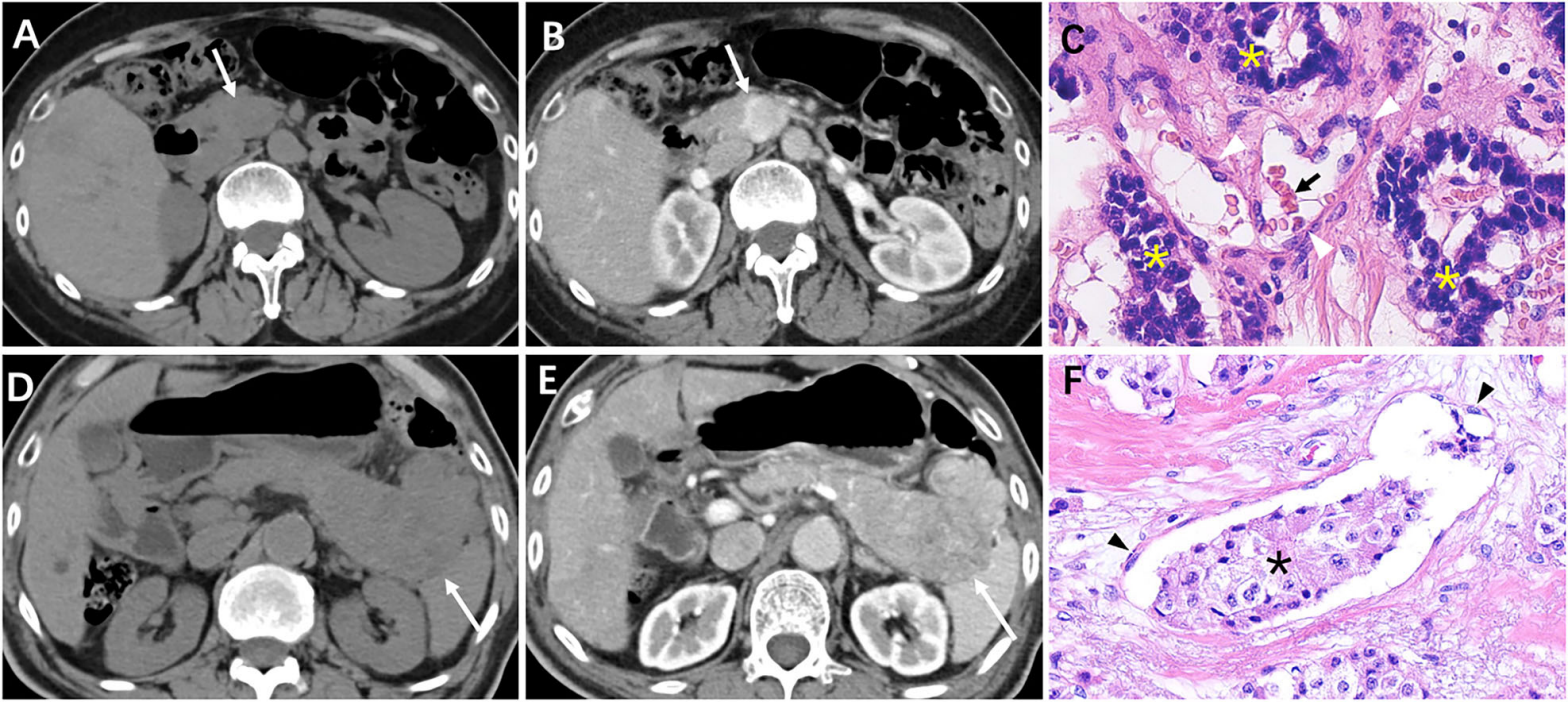
MVI status in patients with PNETs. **A**, **B** A 51-year-old female with grade 1 PNET (without MVI) within the uncinate of the pancreas presents with hypervascular appearance (white arrow). **C** Staining with H&E (× 40) on the slide reveals a grade 1 PNET without the presence of MVI. The microvascular wall is lined with vascular endothelial cells (white arrowhead), while the lumen contains red blood cells (black arrow). Surrounding the microvascular structures are tumor cells characterized by hyperchromatic nuclei (yellow asterisk). **D**, **E** A 77-year-old male with grade 2 PNET (with MVI) located in the body-tail region of the pancreas, presents with heterogeneous enhancement (white arrow). **F** Staining with H&E (× 40) on the slide shows a grade 2 PNET exhibiting the presence of MVI. The microvascular lumen is observed to contain a tumor embolus (black asterisk), while the wall is lined with vascular endothelial cells (black arrowhead)

Table 1 shows the demographic information and radiological characteristics of the training group, and Fig. 2 depicts MVI status in patients. The training group included 160 individuals (mean/median age 54.8/56 years, range 16–81 years), with 25 (15.6%) having MVI and 135 (84.4%) not having MVI. Symptoms were present in 44.4% (71/160) of patients, mainly abdominal pain, followed by dizziness, epigastric discomfort, and fatigue. The MVI group had a median age of 53.0 years (vs. 57.0 years in non-MVI), a higher proportion of females (56.0% vs. 52.6%), and more cases with symptoms (56.0% vs. 42.2%) or elevated tumor markers (24.0% vs. 14.8%), but none showed statistical significance (*p* > 0.05). PNETs with MVI had a significantly larger diameter (median 36.7 mm vs. 23.4 mm) than those without MVI (*p* = 0.001). Among PNETs sized 20–50 mm, 14.5% (10/69) showed MVI, while 33.3% (10/30) of those larger than 50 mm had MVI. MVI was linked to a higher incidence of irregular shape (56.0% vs. 21.5%), tissue invasion (44.0% vs. 7.4%), lymph node metastases (32.0% vs. 7.4%), and liver metastases (32.0% vs. 4.4%) compared to non-MVI cases (all *p* < 0.05). No significant differences were found in tumor location (body/tail, 52.0% vs. 52.6%), pancreatic duct dilatation (12.0% vs. 14.8%), calcification (24.0% vs. 17.0%), or tumor texture (solid, 72.0% vs. 63.7%) (all *p* > 0.05). CT enhanced parameters showed that PNETs with MVI had lower portal lesion enhancement (median 90 vs. 111.0 HU), enhancement ratio (1.02 ± 0.24 vs. 1.23 ± 0.39), absolute enhancement (median 51.0 vs. 73.0 HU), and relative enhancement ratio (median 1.31 vs. 1.85) compared to those without MVI (all *p* < 0.05).

**Table 1:** Demographic information and radiological characteristics of the training cohort

	Non-MVI (*n* = 135)	MVI (*n* = 25)	Adjusted *p*-value*
Age (years)	57.0 (46.0–65.0)	53.0 (42.5–67.0)	0.757
Gender			0.801
Male	64 (47.4)	11 (44.0)	
Female	71 (52.6)	14 (56.0)	
Symptom	57 (42.2)	14 (56.0)	0.345
Tumor marker	20 (14.8)	6 (24.0)	0.391
Location			0.957
Head or neck	64 (47.4)	12 (48.0)	
Body or tail	71 (52.6)	13 (52.0)	
Largest diameter (mm)	23.4 (14.4–37.2)	36.7 (23.5–64.7)	**0.002**
Pancreatic duct dilatation	20 (14.8)	3 (12.0)	0.801
Calcification	23 (17.0)	6 (24.0)	0.555
Irregular shape	29 (21.5)	14 (56.0)	**0.002**
Tumor texture			0.555
Solid	86 (63.7)	18 (72.0)	
Solid and cystic	49 (36.3)	7 (28.0)	
Invasion of surrounding tissues	10 (7.4)	11 (44.0)	**0.002**
Lymph node metastases	10 (7.4)	8 (32.0)	**0.002**
Liver metastases	6 (4.4)	8 (32.0)	**0.002**
CT enhanced parameters			
Portal lesion enhancement	111.0 (93.0–144.0)	90.0 (78.0–105.5)	**0.002**
Enhancement ratio	1.23 ± 0.39	1.02 ± 0.24	**0.017**
Absolute enhancement	73.0 (54.0–104.0)	51.0 (35.0–71.0)	**0.002**
Relative enhancement ratio	1.85 (1.30–2.74)	1.31 (0.83–1.87)	**0.011**

* Adjusted *p*-value was calculated using the Benjamini–Hochberg correction method to control the false discovery rates (FDRs)

Bold values indicate a *p*-value of less than 0.05

### Demographic information and radiological characteristics of the validation group

The clinical and imaging features of the validation group are detailed in Supplementary Material 2. The validation group included 28 patients, with 8 having MVI and 20 not having MVI. PNETs with MVI had larger largest diameter (39.4 mm vs. 19.5 mm), a higher proportion of irregular shape (75.0% vs. 20.0%), invasion of surrounding tissues (75.0% vs. 10.0%), and present with lower portal lesion enhancement (100.3 ± 8.4 vs. 124.8 ± 30.0 HU), enhancement ratio (1.09 ± 0.19 vs. 1.31 ± 0.28), absolute enhancement (median 59.0 vs. 72.0 HU), and relative enhancement ratio (1.49 ± 0.24 vs. 2.08 ± 0.74) compared to those without MVI (all *p* < 0.05). However, lymph node metastases and liver metastases did not show statistical significance as seen in the validation group.

### Comparison between the training and validation groups

The clinical and imaging features of the training and validation groups are detailed in Table 2. The validation group exhibited a higher median age (63.5 vs. 56.0 years) and a greater proportion of male patients (67.9% vs. 46.9%) with significant differences (*p* < 0.05). Lesions in the validation group were more frequently associated with a solid texture (92.9% vs. 65.0%, *p* < 0.05) and invasion of surrounding tissues (28.6% vs. 13.1%, *p* < 0.05). No other factors showed significant differences.

**Table 2:** Demographic information and imaging features between the training and validation groups

	Training (*n* = 160)	Validation (*n* = 28)	*p*-value
MVI	25 (15.6)	8 (28.6)	0.097
Age (years)	56.0 (45.2–65.0)	63.5 (55.0–70.0)	**0.023**
Gender			**0.041**
Male	75 (46.9)	19 (67.9)	
Female	85 (53.1)	9 (32.1)	
Symptom	71 (44.4)	12 (42.9)	0.881
Tumor marker	26 (16.3)	4 (14.3)	0.793
Location			0.421
Head or neck	76 (47.5)	11 (39.3)	
Body or tail	84 (52.5)	17 (60.7)	
Largest diameter (mm)	24.4 (14.9–40.3)	24.3 (14.4–39.7)	0.979
Pancreatic duct dilatation	23 (14.4)	3 (10.7)	0.605
Calcification	29 (18.1)	4 (14.3)	0.622
Irregular shape	43 (26.9)	10 (35.7)	0.338
Tumor texture			**0.003**
Solid	104 (65.0)	26 (92.9)	
Solid and cystic	56 (35.0)	2 (7.1)	
Invasion of surrounding tissues	21 (13.1)	8 (28.6)	**0.037**
Lymph node metastases	18 (11.3)	2 (7.1)	0.516
Liver metastases	14 (8.8)	4 (14.3)	0.358
CT enhanced parameters			
Portal lesion enhancement	109.0 (90.0–139.0)	109.0 (97.3–129.8)	0.665
Enhancement ratio	1.19 ± 0.37	1.25 ± 0.27	0.809
Absolute enhancement	66.0 (51.0–101.0)	67.0 (60.0–86.5)	0.520
Relative enhancement ratio	1.69 (1.20–2.66)	1.71 (1.49–2.26)	0.620

Bold values indicate a *p*-value of less than 0.05

### Interobserver agreement and Pearson correlation analysis

The interobserver agreement on the selected parameters was excellent, with stable ICC values, which was listed as follows: portal lesion enhancement, 0.987 (95% CI, 0.972–0.994); enhancement ratio, 0.989 (95% CI, 0.960–0.991); absolute enhancement, 0.982 (95% CI, 0.960–0.991); relative enhancement ratio, 0.963 (95% CI, 0.920–0.983).

The correlation matrix between the largest diameter and enhancement variables is provided in Supplementary Material 3. Significant differences were observed between the largest diameter and both portal lesion enhancement and absolute enhancement, with *p*-values of 0.008 and 0.011, and correlation coefficients of −0.21 and −0.20, respectively. However, no significant differences were found between the largest diameter and enhancement ratio or relative enhancement ratio, with *p*-values of 0.137 and 0.055, and correlation coefficients of −0.12 and −0.15, respectively.

### Logistic regression analysis

**Fig. 3:**
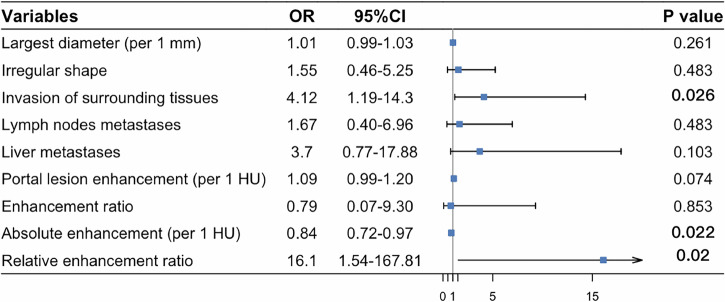
Forest map confirmed through univariate and multivariate logistic regression analyses. From this plot, we found that invasion of surrounding tissues (OR: 4.12, 95% CI, 1.19–14.3), absolute enhancement (OR: 0.84, 95% CI, 0.72–0.97), and relative enhancement ratio (OR: 16.1, 95% CI, 1.54–167.81) were identified as independent predictors for predicting MVI in PNET patients

Figure 3 presents the multivariable logistic analysis results as a forest plot based on the training group. Factors with *p* < 0.05 from the univariable analysis were included: tumor diameter, irregular shape, tissue invasion, lymph node metastases, liver metastases, portal lesion enhancement, enhancement ratio, absolute enhancement, and relative enhancement ratio. Multivariable analysis identified three independent predictors for MVI in PNETs: invasion of surrounding tissues (OR: 4.12, 95% CI, 1.19–14.3), absolute enhancement (OR: 0.84, 95% CI, 0.72–0.97), and relative enhancement ratio (OR: 16.1, 95% CI, 1.54–167.81) (all *p* < 0.05).

### Diagnostic performance

**Fig. 4:**
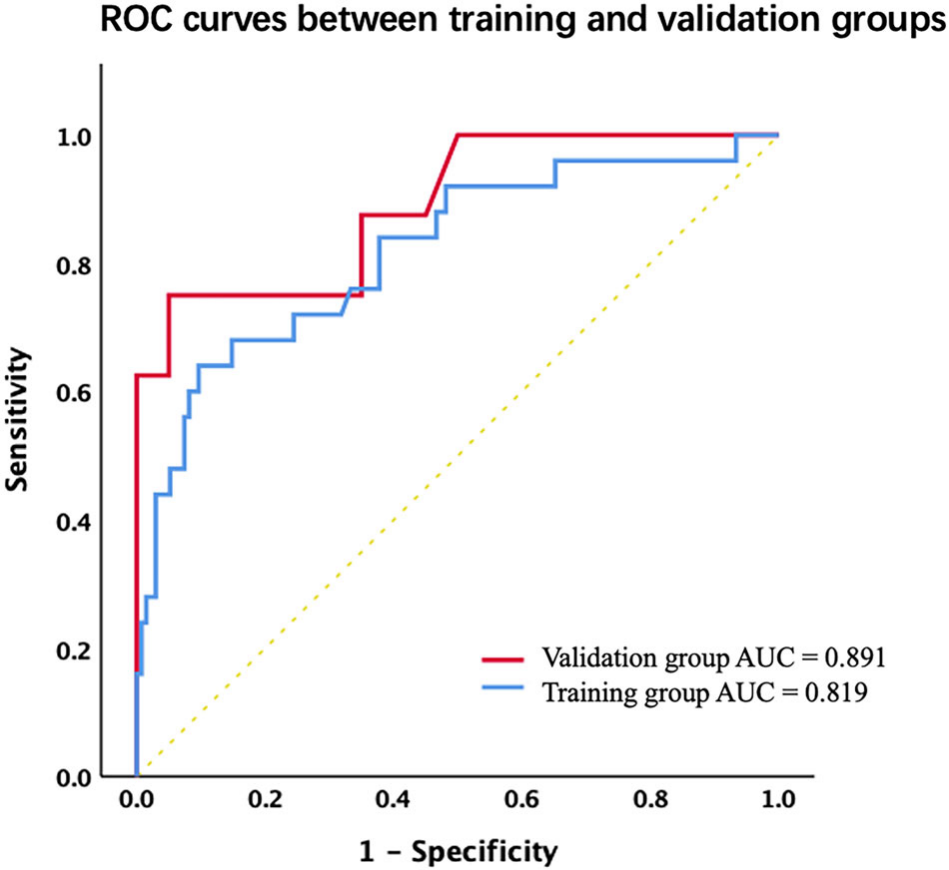
Receiver operating characteristic curve of the predictive model both in the training and validation groups. The model achieved an AUC of 0.819 (95% CI, 0.720–0.917), demonstrating a sensitivity of 64% and a specificity of 90.4% in the training group. The AUC of the validation group was 0.891 (95% CI, 0.751–1.0), with a sensitivity of 87.5%, and a specificity of 65%

Figure 4 shows the ROC curves for predictive performance in the training and validation groups. The training group model had an AUC of 0.819 (95% CI, 0.720–0.917), with 64% sensitivity and 90.4% specificity. The validation group AUC was 0.891 (95% CI, 0.751–1.0), with 87.5% sensitivity and 65% specificity.

For absolute enhancement ≤ 80 HU, the sensitivity for diagnosing a PNET with MVI was 88%, while the specificity was only 43.7%; when its value was > 150 HU, sensitivity dropped to 0% and specificity rose to 94.1%. For relative enhancement ratio ≤ 200% (same as 2.0), sensitivity was 80% and specificity 43%; for > 400%, sensitivity was 0% and specificity 93.3%.

### Shiny app establishment

**Fig. 5:**
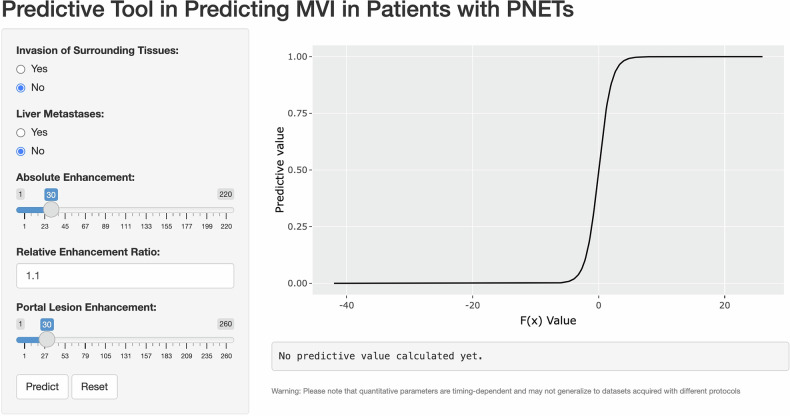
Predictive tool in predicting MVI in patients with PNETs in R/shiny app. An illustrative example is provided to demonstrate the functionality of this tool. Physicians simply input relevant values, and the predictive outcome is generated. In this specific instance, the calculated chance of MVI occurrence in the patient is minimal, with only a 1.671% probability

Figure 5 displays the graphical user interface of the predictive tool for MVI in PNET patients. The graphical user interface is available online (https://xmfchy.shinyapps.io/shinyapp/). The formula from multivariable logistic regression is: f(x) = 1.84* invasion of surrounding tissues + 1.961* liver metastases − 0.165* absolute enhancement + 2.536 * relative enhancement ratio + 0.081 * portal lesion enhancement − 4.490. The probability predictive value for MVI in patients with PNETs was calculated as follows: exp(f(x)) / (1 + exp(f(x))). The optimal cutoff value for predicting MVI was 0.304, with a sensitivity of 0.64 and a specificity of 0.904. The web-based R/shiny app we have developed offers an intuitive and user-friendly interface, allowing users to effortlessly select or input the relevant values. Subsequently, the app calculates the predictive value to determine the probability of MVI in patients with PNETs.

### Survival outcomes

**Fig. 6:**
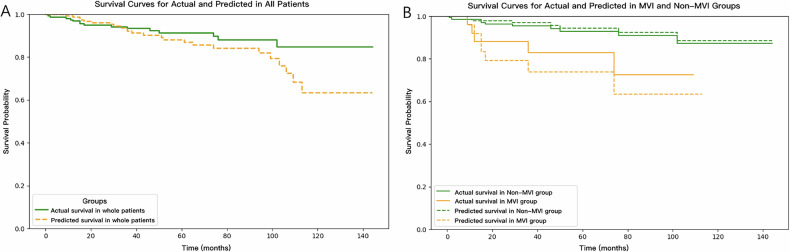
The Kaplan–Meier curves illustrate the actual and predicted overall survival of patients with PNETs in the entire cohort and the MVI subgroups. The dashed line represents the predicted survival curves

Figure 6 shows Kaplan–Meier curves illustrating the actual and predicted overall survival of patients with PNETs in the entire cohort and the MVI subgroups. In the training group, the median overall survival for PNET patients was 52.5 months (range 1–144 months) with an average of 61 months. Patients with MVI had a median survival of 12 months, compared to 37.5 months for those without MVI (log-rank *p* = 0.034), indicating poorer outcomes for MVI patients. A similarly significant difference was found in the predicted overall survival between the two groups (log-rank *p* < 0.001). The actual survival probabilities at 12 months were 88.0% for the MVI group and 98.5% for the Non-MVI group, while at 36 months, they were 82.8% and 95.4%, respectively. The predicted survival probabilities at 12 months were 91.7% for the MVI group and 97.8% for the Non-MVI group, and at 36 months, they were 73.9% and 96.9%, respectively. For the entire cohort, predicted and actual survival showed no significant difference (log-rank test: *p* = 0.053). However, a significant difference was observed in the MVI group (*p* = 0.008), while no difference was found in the non-MVI group between the predicted and actual survival curves (*p* = 0.221).

## Discussion

Currently, the prediction of MVI in patients with PNETs has received relatively less attention, despite the strong correlation between MVI and tumor heterogeneity as well as prognosis [9, 10]. We used clinical and radiological features from portal venous phase images to predict MVI before surgery and compare survival outcomes. Multivariable logistic regression identified invasion of surrounding tissues, absolute enhancement, and relative enhancement ratio as independent predictors for MVI in PNETs. The predictive model achieved an AUC of 0.819 and 0.891, with sensitivities of 64% and 87.5%, and specificities of 90.4% and 65% in the training and validation groups, respectively, indicating favorable performance. A web-based R/shiny app for MVI prediction was developed, providing real-time data-driven estimates for clinical decision-making. MVI was associated with worse overall survival compared to non-MVI cases, both in actual and predicted overall survival. Our study is a pioneering effort in using imaging features to develop a user-friendly predictive tool for preoperative MVI prediction, offering insights into tumor heterogeneity.

The past decade has witnessed a remarkable advancement in the understanding of PNETs’ physiology, the performance of their heterogeneity, and the modalities used for their treatment [1, 25, 29]. CT scans have long been crucial for diagnosing PNETs, revealing both evident features (size, shape, texture) and subtle characteristics (enhancement patterns, especially in the portal venous phase) [30]. In our study, we focused on tumor enhancement features during the portal venous phase and found significant differences between PNETs with and without MVI. PNETs with MVI showed notably reduced enhancement patterns, likely due to microvascular involvement causing small blood vessel occlusion and reduced blood supply. Both absolute enhancement and relative enhancement ratio were identified as independent risk factors for predicting MVI, suggesting that intra-tumoral characteristics may better reflect tumor angiogenesis than overall tumor features.

Park’s study showed that the tumor-to-parenchymal ratio (portal enhancement ratio) on the portal phase had superior predictive performance for postoperative survival compared to current grading and staging systems for PNETs, highlighting its significant impact on outcomes beyond tumor extent [22]. The results of another study also indicated that the portal enhancement ratio ≤ 1.1 served as a prognostic imaging biomarker for predicting worse survival outcomes in patients with PNETs [24]. A lower enhancement ratio indicates reduced tumor blood flow, which significantly correlates with a high proliferative index, suggesting a higher tumor grade [31]. In our study, MVI, irregular shape, invasion of surrounding tissues, and metastases were linked to worse overall survival. MVI’s presence indicated poor prognosis, and identifying enhancement factors related to MVI may lead to new prognostic markers. Another study found that a portal enhancement ratio < 1.02 and lymph node metastases were independent factors for poorer survival outcomes in PNET patients [16]. The relative enhancement ratio in the portal venous phase could accurately discriminate lipid-poor adenomas from non-adenomas, indicating its good diagnostic performance [27]. In our previous study, we used quantitative parameters from enhanced CT scans to differentiate well-differentiated from poorly differentiated PNETs. We found that both arterial and portal absolute enhancements were independent predictors with strong diagnostic performance, reflecting important biological characteristics like tumor differentiation, grade, and proliferative index [13]. The combination of enhancement features and involvement of the main pancreatic duct were reliable predictors for predicting PNETs with high-risk histological factors [11].

Qualitative imaging features, along with enhancement parameters, are crucial for predicting MVI. Our study found that PNETs with MVI often showed irregular shapes, invasion of surrounding tissues, and metastases, indicating a link between MVI and poorer survival outcomes. Zhang’s study [9] found that MVI and tumor grade were independent prognostic variables for prediction of overall survival. Meanwhile, the presence of MVI was also identified as an independent risk factor for predicting recurrence in patients with PNETs, compared to other pathological features, such as lymphatic and perineural invasion [10]. Invasion of adjacent tissues was a strong predictor for MVI in PNET patients. However, we did not separately analyze larger venous invasion and nearby tissues/organs, requiring further research into the specific link between macrovascular invasion and MVI. In Addeo et al [32] study, they discovered that macrovascular venous invasion was more common, demonstrated with perineural invasion, larger size, lymph node involvement and poorly differentiated tumors. MVI was also associated with lymph node metastases, alongside tumor size larger than 2 cm, distant metastases, and perineural invasion in another study [17].

Creating a diagnostic or predictive model is not inherently difficult, but translating these models into practical, user-friendly clinical applications is challenging. The Shiny app addresses this by providing an intuitive interface for exploring complex medical data and aiding decision-making [33]. In our study, the R/Shiny app’s interface included five factors, allowing real-time calculation of MVI probability for PNET patients, making it convenient for clinical practice. The shiny app could also aid oncologists in identifying precision clinical trials [34] and predicting hepatic steatosis for selecting optimal living donors [35]. As technology advances, the shiny app could improve diagnostics, treatment planning, and patient care through its interactive and accessible interface.

The study has several limitations. First, diverse CT protocols were implemented due to the long period of data collection, which may potentially introduce measurement deviations, while the retrospective design may lead to selection bias. Second, since not all images were reconstructed at a 1.25 mm slice thickness, the use of 3–5 mm slices for assessing vascular and adjacent tissue involvement may have introduced some inaccuracies in the evaluation. Third, the portal venous phase was acquired at approximately 60 s post-contrast injection rather than the conventional 70 s, which may yield different enhancement values and affect the model’s performance. Fourth, the validation group, though included, had a small sample size, limiting its reliability. Fifth, only survival data from the training group were collected, due to the short follow-up time of the validation group.

In conclusion, invasion of surrounding tissues, absolute enhancement, and relative enhancement ratio were identified as independent predictors for predicting MVI in patients with PNETs. Patients with MVI have worse overall survival compared to those without MVI. Furthermore, clinical and imaging features from portal venous phase CT images can be used to accurately predict the presence of MVI in patients with PNETs prior to surgery. In real clinical practice, the web-based R/shiny app for predicting MVI provides real-time data-driven estimates of predictive value to facilitate informed decision-making through its interactive and accessible interfaces.

## Supplementary information

ELECTRONIC SUPPLEMENTARY MATERIAL

## Data Availability

The datasets generated and/or analyzed during the current study are not publicly available because the cases are from the Picture Archiving and Communication System of our Hospitals, but are available from the corresponding author upon reasonable request.

## Abbreviations

AUC: Area under the curve
CI: Confidence interval
G1/2/3: Grade1/2/3
HU: Hounsfield unit
ICC: Intraclass correlation coefficient
MVI: Microvascular invasion
NECs: Neuroendocrine carcinomas
OR: Odds ratio
PNETs: Pancreatic neuroendocrine tumors
ROI: Region of interest
ROC: Receiver operating characteristic
