# Supplementary material for: Preoperative prediction of microvascular invasion in pancreatic neuroendocrine tumors through analysis of portal venous phase CT images
Source: Insights Imaging. 2025 Sep 25;16:206. doi: 10.1186/s13244-025-02091-6 (PMC12463792; doi:10.1186/s13244-025-02091-6)

Preoperative Prediction of Microvascular Invasion in Pancreatic Neuroendocrine Tumors Through Analysis of Portal Venous Phase CT Images

ELECTRONIC SUPPLEMENTARY MATERIAL

Supplementary material 1. The list of multiple CT scanners.

Multiple CT scanners were sued as follows:

Siemens Healthcare: Somatom Definition Perspective, Flash 64, AS 6;

GE Healthcare: Optima CT680 Series, BrightSpeed 16;

TOSHIBA Medical Systems Corporation: TOSHIBA Aquilion 320;

Philips Medical Systems: Ingenuity CT 64;

United Imaging Healthcare Technology Company: UIH uCT 760.

Supplementary material 2. Demographic information and radiological characteristics of the validation cohort.

	Non-MVI (n=20)	MVI (n=8)	p-value
Age (years)	62.3 ± 11.9	56.3 ± 13.4	0.287
Gender			0.337
Male	12 (60.0)	7 (87.5)	
Female	8 (40.0)	1 (12.5)	
Symptom	6 (30.0)	6 (75.0)	0.080
Tumor marker	2 (10.0)	2 (25.0)	0.669
Location			1.0
Head or neck	8 (40.0)	3 (37.5)	
Body or tail	12 (60.0)	5 (62.5)	
Largest diameter (mm)	19.5 (12.9-34.6)	39.4 (26.3-57.7)	**0.043**
Pancreatic duct dilatation	19 (95.0)	6 (75.0)	0.385
Calcification	2 (10.0)	2 (25.0)	0.669
Irregular shape	4 (20.0)	6 (75.0)	**0.021**
Tumor texture			1.0
Solid	19 (95.0)	7 (87.5)	
Solid and cystic	1 (5.0)	1 (12.5)	
Invasion of surrounding tissues	2 (10.0)	6 (75.0)	**0.003**
Lymph nodes metastases	1 (5.0)	1 (12.5)	1.0
Liver metastases	1 (5.0)	3 (37.5)	0.105
**CT enhanced parameters**			
Portal lesion enhancement	124.8 ± 30.0	100.3 ± 8.4	**0.003**
Enhancement ratio	1.31 ± 0.28	1.09 ± 0.19	**0.026**
Absolute enhancement	72.0 (63.5-92.0)	59.0 (55.8-64.3)	**0.015**

Relative enhancement ratio	2.08 ± 0.74	1.49 ± 0.24	**0.004**

Supplementary material 3. The correlation matrix between the largest diameter and enhancement variables.

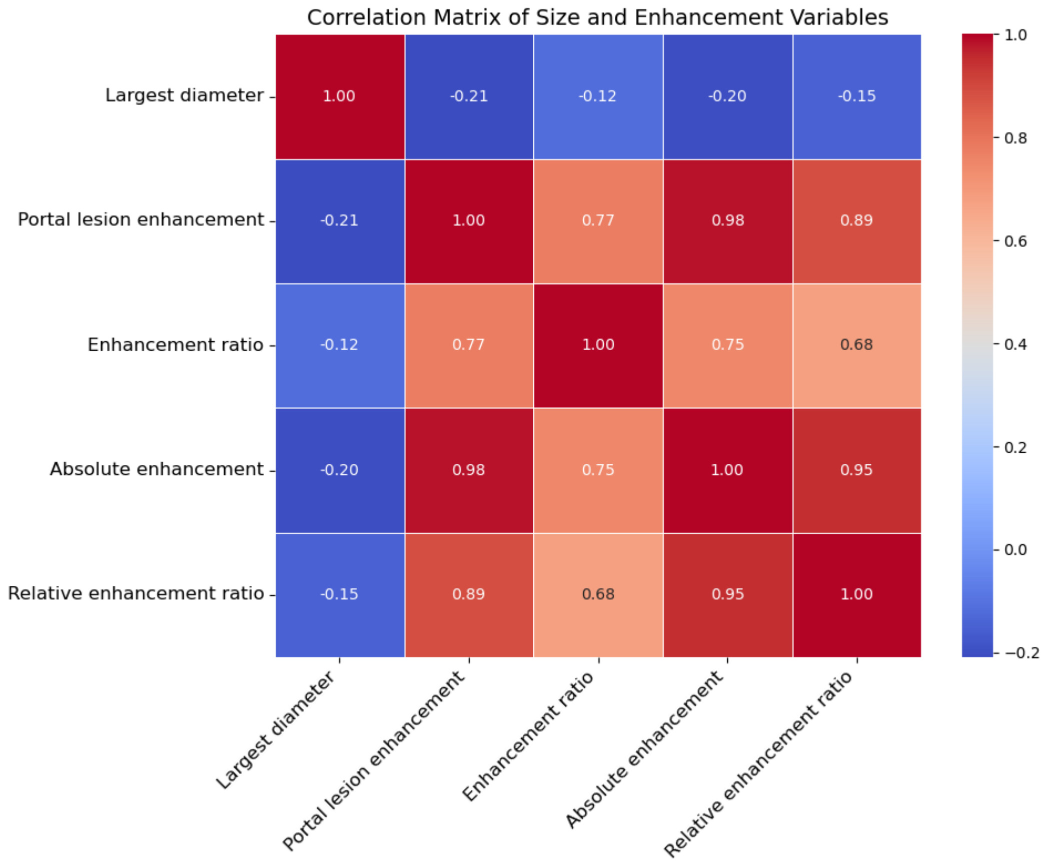

Supplement: Supplementary file 1 — ELECTRONIC SUPPLEMENTARY MATERIAL [file 13244_2025_2091_MOESM1_ESM.pdf]
